# Therapeutic approaches to CNS diseases via the meningeal lymphatic and glymphatic system: prospects and challenges

**DOI:** 10.3389/fcell.2024.1467085

**Published:** 2024-09-06

**Authors:** Rui Zhang, Jiuhong Li, Xueying Li, Si Zhang

**Affiliations:** Department of Neurosurgery, Department of Cardiovascular Surgery, West China Hospital, Sichuan University, Chengdu, China

**Keywords:** meningeal lymphatic vessels, glymphatic system, CNS diseases, drug delivery, neurodegenerative diseases

## Abstract

The brain has traditionally been considered an “immune-privileged” organ lacking a lymphatic system. However, recent studies have challenged this view by identifying the presence of the glymphatic system and meningeal lymphatic vessels (MLVs). These discoveries offer new opportunities for waste clearance and treatment of central nervous system (CNS) diseases. Various strategies have been developed based on these pathways, including modulation of glymphatic system function, enhancement of meningeal lymphatic drainage, and utilization of these routes for drug delivery. Consequently, this review explores the developmental features and physiological roles of the cerebral lymphatic system as well as its significance in various CNS disorders. Notably, strategies for ameliorating CNS diseases have been discussed with a focus on enhancing glymphatic system and MLVs functionality through modulation of physiological factors along with implementing pharmacological and physical treatments. Additionally, emphasis is placed on the potential use of the CNS lymphatic system in drug delivery while envisioning future directions in terms of mechanisms, applications, and translational research.

## 1 Introduction

**FIGURE 1:**
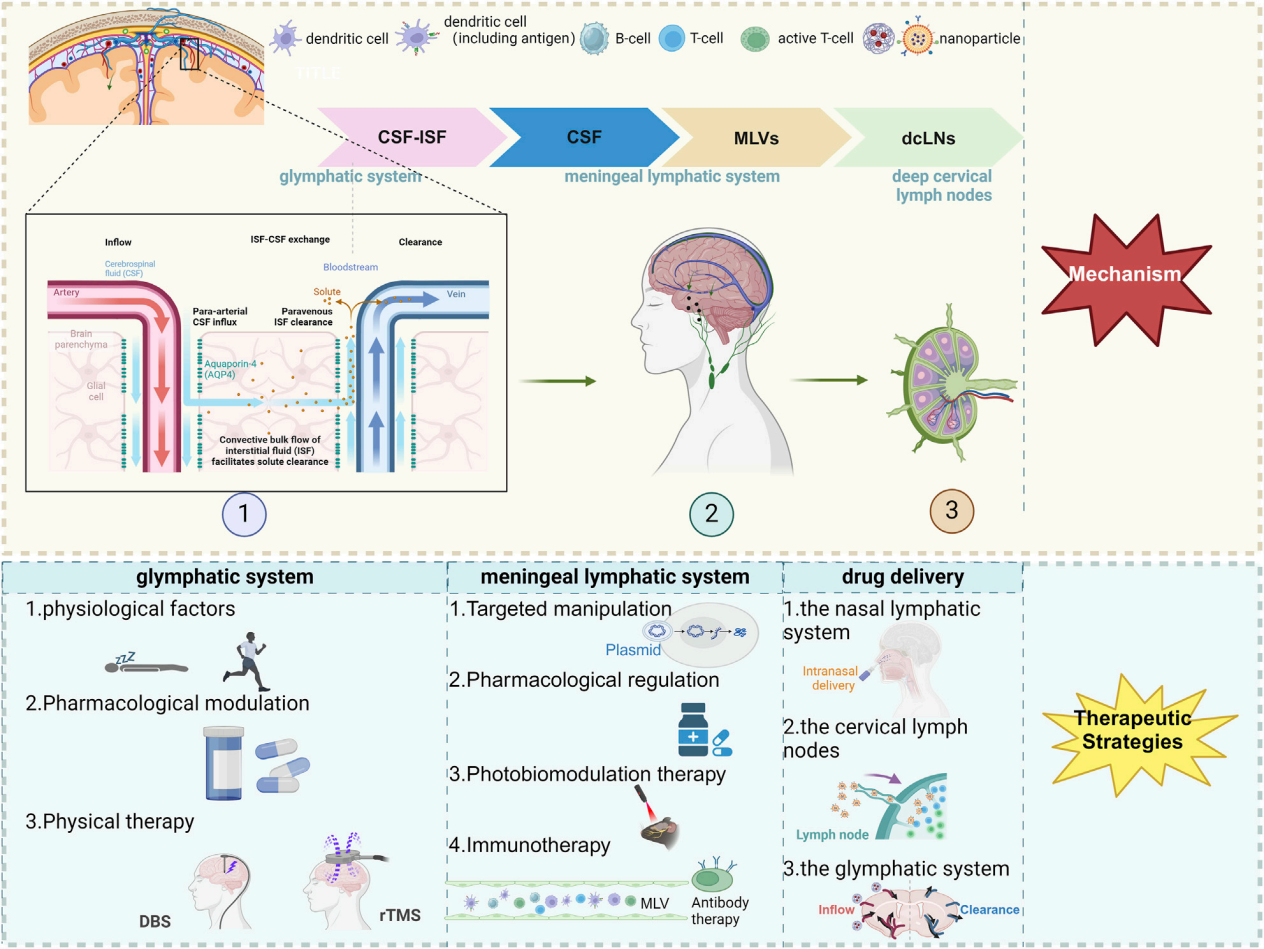
The scheme of therapeutic approaches to CNS diseases via the meningeal lymphatic and glymphatic systems. Created with Biorender.com. Abbreviations: CSF, cerebrospinal fluid; ISF, interstitial fluid; MLVs, meningeal lymphatic vessels; dcLNs, deep cervical lymph nodes; AQP4, Aquaporin-4; DBS, deep brain stimulation; rTMS, repetitive Transcranial Magnetic Stimulation.

**FIGURE 2:**
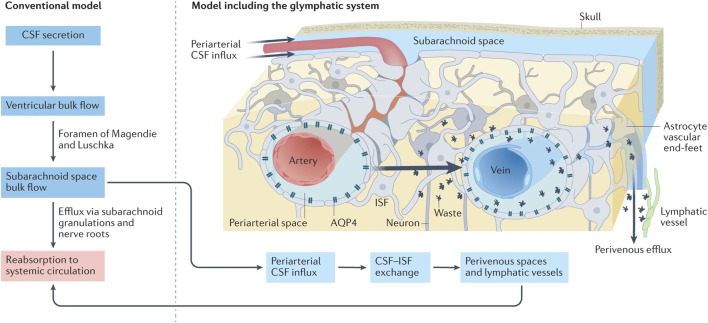
Under traditional CSF drainage patterns (left), the CSF, producing in the ventricular arachnoid mater, circulates within the subarachnoid space before ultimately being reabsorbed back into the blood circulation. In the emerging CSF drainage model under the participation of the glymphatic system (right), the CSF can access the brain parenchyma via perivascular spaces surrounding the arteries, subsequently exchange with the ISF, which infiltrates into the subarachnoid space containing CSF via perivascular spaces surrounding the veins (reproduced from Lohela et al., 2022, with permission from Springer Nature). Abbreviations: CSF, cerebrospinal fluid; ISF, interstitial fluid.

The lymphatic system plays a vital role in transporting interstitial fluid (ISF) from various tissues back to circulation, facilitating immune surveillance and response, as well as maintaining fluid balance (Oliver et al., 2020). Recent studies have provided evidence for its multifunctional role and positive influence on organ-specific physiological functions and disease processes (Petrova and Koh, 2020). The brain, previously believed to lack traditional lymphatic vessels, is considered an organ with unique immune privilege (Louveau et al., 2015a). Consequently, the mechanisms employed by the brain to eliminate the metabolic waste generated by cerebral tissues remain unclear, and this clearance process significantly contributes to the pathogenesis, progression, and prognostic assessment of central nervous system (CNS) diseases. Interestingly, over two centuries ago, Paolo Mascagni described the existence of meningeal lymphatic vessels (MLVs), but his findings were overlooked due to contradicting mainstream beliefs at that time (Sandrone et al., 2019). In 2012, Iliff et al. (2012) discovered an internal cerebrospinal exchange and clearance pathway that relies on the Aquaporin-4 (AQP4) protein located at the astrocytic endfeet and penetrating vessel perivascular space. Through this route, similar to that of peripheral lymphatic system function, large molecules along with pathological proteins and interstitial solutes exit the brain parenchyma into cerebrospinal fluid (CSF) (Iliff et al., 2012). Given its similarity in mechanism with the peripheral lymphatic system, it was appropriately named the glymphatic system by the scientific community (Louveau et al., 2015a) (Figures 1, 2).

**FIGURE 3:**
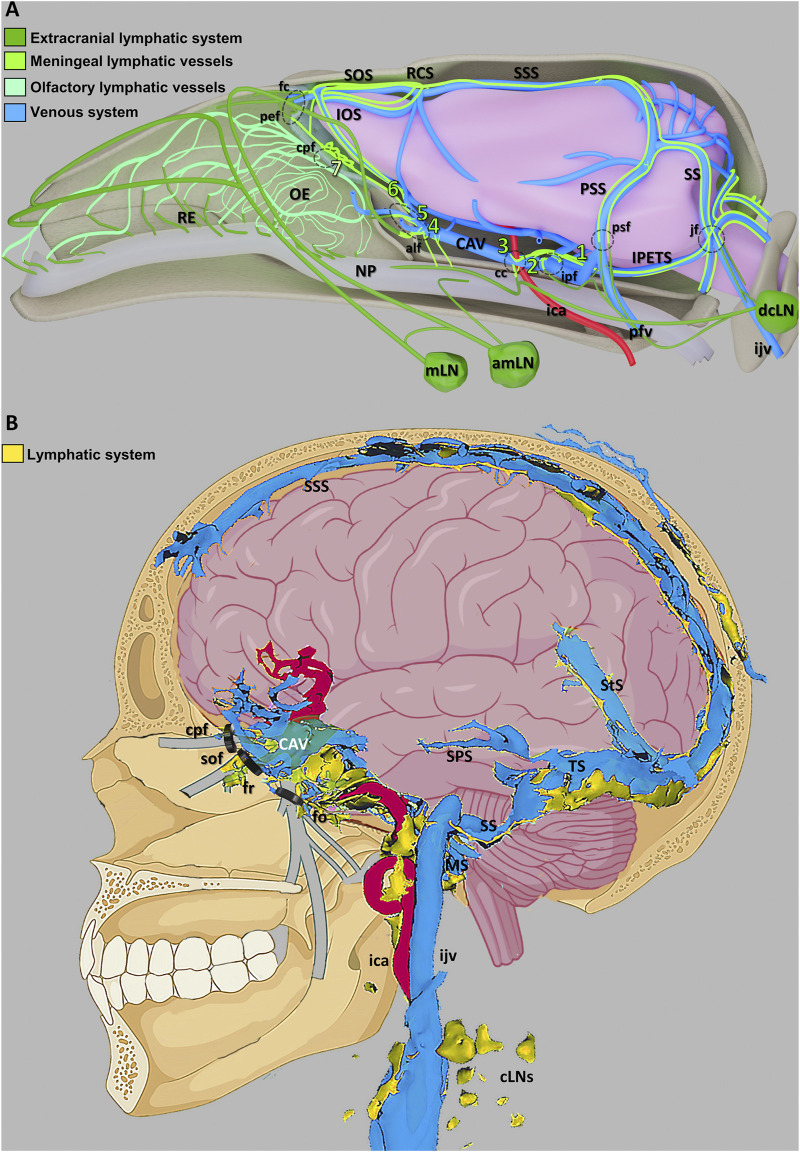
Schematic illustration of the CNS venous and lymphatic systems in mice and humans **(A)** A summary of the mouse head’s lymphatic CSF draining circuitry. **(B)** Diagram showing the human dural venolymphatic complex with lymphatic discharges (reproduced from Jacob et al., 2022, licensed under CC BY 4.0). Abbreviations: RE, respiratory epithelium; OE, olfactory epithelium; NP, nasopharynx; mLN, mandibular lymph node; amLN, accessory mandibular LN; IOS, inferior olfactory sinus; SOS, superior olfactory sinus; RCS, rostral confluence of sinuses; CAV, cavernous sinus; SSS, superior sagittal sinus; PSS, petrosquamous sinus; SS, sigmoid sinus; IPETS, inferior petrosal sinus; dcLN, deep cervical LN; sfc, foramen caecum; pef, posterior ethmoid foramen; cpf, cribriform plate foramina; alf, anterior lacerated foramen; cc, carotid canal; ipf, interpterygoid foramen; ica, internal carotid artery; pfv, posterior facial vein; psf, petrosquamous fissure; jf, jugular foramen; ijv, internal jugular vein; SPS, superior petrosal sinus; StS, straight sinus; TS, transverse sinus; SS, sigmoid sinuses; MS, marginal sinus; cLNs, cervical LNs; sof, superior orbital fissure; fr, foramen rotundum; fo, foramen ovale.

In 2015, research conducted by Aspelund et al. (2015); Louveau et al. (2015b) revealed the existence of MLVs in the dorsal and basal regions of the brain, which are lined with fully differentiated lymphatic endothelial cells (LECs) resembling classic lymphatic vessels found in peripheral tissues. These MLVs express lymphatic markers such as Vascular Endothelial Growth Factor Receptor 3 (VEGFR3) and Prospero-Related Homeobox Protein 1 (PROX1), challenging the conventional notion of cerebral immune “privilege.” Subsequently, scientists have further substantiated the presence of MLVs at the skull base within the meninges, consistent with Aspelund et al. (2015) findings, and revealed that these basal MLVs possess lymphatic valves (Ahn et al., 2019). Furthermore, MLVs were identified as conduits for CSF drainage into deep cervical lymph nodes (dcLNs) (Louveau et al., 2015b). High-resolution magnetic resonance imaging (MRI) has been employed to demonstrate the presence of MLVs in both humans and non-human primates (Absinta et al., 2017). More interestingly, researchers utilized light-sheet fluorescence microscopy and MRI to achieve comprehensive whole-head imaging of intact MLVs in mice and humans alike (Figure 3) (Da Mesquita, 2022; Jacob et al., 2022). Collectively, these findings provide compelling evidence for the existence of a functional CNS lymphatic system.

Additionally, the recently discovered CNS lymphatic system exhibits crucial physiological functions in eliminating metabolic waste from the brain and regulating immunity (Rasmussen et al., 2022). Dysfunction of this system have been observed in various CNS diseases (Rasmussen et al., 2022). This review outlines both developmental features and physiological functions of the CNS lymphatic system while also discussing its implications in different diseases. Furthermore, it emphasizes strategies for intervening in brain diseases by targeting the CNS lymphatic system, specifically focusing on modulating the glymphatic system, manipulating MLVs, and utilizing drug delivery mediated by the CNS lymphatic system to achieve therapeutic objectives for CNS diseases. Finally, this article concludes with a summary of strategies involving the CNS lymphatic system for treating CNS diseases and provides insights into potential directions for future research (Figure 1).

## 2 Developmental features, physiological functions, and disease associations of the CNS lymphatic system

### 2.1 Developmental features of the CNS lymphatic system

The glymphatic system is not a lymphatic structure comprising fully matured LECs (Iliff et al., 2012). However, owing to its similar functionality as peripheral lymphatic vessels in promoting the clearance and drainage of metabolic waste from the ISF of the brain, it is referred to as the glymphatic system. Its primary constituents include astrocytes and their end-feet protein, AQP4 (Iliff et al., 2012), along with perivascular spaces known as Virchow-Robin spaces (VRS) (Rennels et al., 1985). Astrocytes mainly play a supportive role by adhering to the penetrating vessels of the brain. Meanwhile, AQP4 is expressed on their surface, enabling the delivery of nutrient-rich CSF into the brain parenchyma for nourishing neurons, glial cells, and other neurocytes (Plog and Nedergaard, 2018). The perivascular spaces encompass both arteriolar and venular perivascular spaces that serve as conduits for the inflow of CSF and outflow of ISF (Rasmussen et al., 2018). Penetrating intracerebral arteries traverse these spaces to reach deep into blind-ended regions of brain parenchyma. Facilitated by the action of AQP4, CSF can follow these spaces and infiltrate extensively within the brain parenchyma. This pathway also provides an opportunity for intrathecally injected drugs in CSF to access deep regions of brain parenchyma (Lohela et al., 2022).

The development of MLVs is characterized by multiple facets. After birth, MLVs develop and extend in synchrony with the growth direction of nerves and blood vessels (Antila et al., 2017). By utilizing MRI techniques, non-invasive visualization and quantification of MLVs, along with studying the flow dynamics within the human MLVs, have been successfully accomplished (Kim et al., 2023). Preliminary results indicate that the flow velocity in dorsal MLVs ranges from 2.2 to 2.7 mm per second in humans. Moreover, a gender discrepancy in MLV volumes is apparent between males and females, with male MLV volumes significantly exceeding those of females (Jacob et al., 2022). In both mice and humans, the drainage capacity of MLVs diminishes with age (Da Mesquita et al., 2018; Albayram et al., 2022). Remarkably, the MLVs exhibited plasticity and regenerative capacity. An increase in Vascular Endothelial Growth Factor C (VEGF-C), a positive factor promoting lymphatic vessel development, induces the sprouting, development, and maintenance of MLVs. Conversely, silencing or blocking VEGF-C signaling leads to noticeable degradation of MLVs, whereas the knockout of Vascular Endothelial Growth Factor D (VEGF-D) does not significantly impact them (Antila et al., 2017). Basal MLVs, adjacent to the subarachnoid space (SAS), possess lymphatic valves akin to peripheral lymphatic vessels and serve as “hotspot” sites for clearing large molecules within CSF (Ahn et al., 2019).

### 2.2 Physiological functions of the CNS lymphatic system

While there is no direct anatomical connection between the glymphatic system and the meningeal lymphatic system, they functionally interact to collectively form a unique network responsible for waste clearance and immune regulation in the brain (Louveau et al., 2015b). In terms of waste clearance, these systems collaboratively establish a CSF drainage pathway: CSF-ISF-CSF-MLV-dcLNs (Li G. et al., 2022). Iliff et al. (2012) demonstrated that the majority of the CSF in the SAS infiltrates into the brain parenchyma. As shown in Figure 2, CSF flows into the brain parenchyma through perivascular spaces surrounding pia mater arteries and penetrating arterioles, where it undergoes mixing as well as nutrients and metabolic waste exchange with ISF . ISF, loaded with interstitial solutes, is expelled from the brain parenchyma via venous perivascular routes (intracerebral and caudal rhinal veins) and eliminated into the CSF (Iliff et al., 2012).

The dural lymphatic vessels then uptake absorb ISF and CSF from the SAS, subsequently transporting them to the dcLNs (Aspelund et al., 2015; Louveau et al., 2015b) (Figure 3). The principal site of absorption is identified as the MLVs located at the base of the skull (Ahn et al., 2019). This discovery offers a novel perspective on the clearance of large molecular solutes in neurodegenerative disorders. Furthermore, CSF is drained through multiple pathways. Traditionally, it is posited that arachnoid granulations facilitate the absorption of CSF from the SAS via a valve-like mechanism, allowing CSF to enter and ultimately be absorbed by the venous sinuses (Welch and Friedman, 1960; Wolpow and Schaumburg, 1972) (Figure 2). Additionally, the arachnoid cuff exit points, formed by discontinuities as the bridging veins traverse the arachnoid barrier, create openings that permit the exchange of CSF and molecules between the SAS and the dura mater, facilitating CSF drainage (Smyth et al., 2024). Recently, the nasopharyngeal lymphatic plexus (NPLP) has been demonstrated to serve as a major hub for the outflow of CSF to the dcLNs (Yoon et al., 2024). Research indicates that the NPLP is connected with upstream lymphatic vessels, including those near the pituitary gland and the cavernous sinus, as well as through the cribriform plate, where CSF is drained to the dcLNs via the deep cervical lymphatic vessels. These CSF drainage structures collaborate with the glymphatic system and the meningeal lymphatic system to jointly sustain the balance of cerebral fluids and the clearance of metabolic waste.

On the other hand, both the glymphatic system and the meningeal lymphatic system play crucial roles in immune cell drainage and immunosurveillance, functioning synergistically (Li G. et al., 2022). Prior to the discovery of the brain lymphatic system, research on neuroimmunity primarily focused on microglia. However, with the identification of this system, it has revitalized studies in neuroimmunology (Sun et al., 2018). Research indicates that antigens generated within the CNS can travel through the glymphatic pathway to reach the SAS before draining into MLVs or passing through the cribriform plate into nasal lymphatics and eventually reaching cervical lymph nodes (cLNs). This process stimulates adaptive immune responses (Louveau et al., 2015a; Louveau et al., 2015b). Mechanistically, neuroinflammation can induce the formation of MLVs adjacent to the cribriform plate. These newly formed lymphatic vessels containing CD4^+^ T cells and CD11c+ cells capable of capturing CNS antigens and presenting them to T cells (Hsu et al., 2022). Additionally, a substantial number of immune cells are located around the meningeal sinuses which serve as an ecological niche known as a “regional immune center.” These cells collaborate with MLVs to perform neuroimmunosurveillance (Rustenhoven et al., 2021; Smyth et al., 2024). Studies have demonstrated that CNS antigens present in the CSF reside around dural sinuses, where they are presented by antigen presenting cells associated with dura mater for patrolling T cells from venous sinuses. These activated T cells migrate through interstitial spaces of MLV endothelial cells into MLVs and are subsequently drained towards cLNs, thereby inducing the generation of T cell effector functions and promoting tissue retention (Rustenhoven et al., 2021). Therefore, MLVs provide a feasible physical conduit for the interaction between CNS antigens and peripheral T cells.

### 2.3 CNS diseases associations of the CNS lymphatic system

Several studies have highlighted dysfunction in CNS lymphatic drainage across a range of brain diseases (Table 1). For instance, in models of subarachnoid hemorrhage (SAH), the glymphatic system is obstructed by fibrin clot blockages in perivascular spaces, thereby impeding CSF inflow into the brain parenchyma (Pu et al., 2019). In cytotoxic brain edema, hypoxia-induced energy depletion results in glymphatic system dysfunction, promoting CSF inflow into the brain parenchyma while inhibiting ISF outflow (Thrane et al., 2014). In the meningeal lymphatic system of idiopathic Parkinson’s disease (PD) patients, a reduction in MLV flow was observed in the superior sagittal sinus and sigmoid sinus regions, along with delayed CSF drainage in deep lymph nodes (Ding et al., 2021), and impaired clearance of α-synuclein (α-syn) by CNS lymphatics (Zou et al., 2019). These findings provide evidence of MLV dysfunction in PD patients. Moreover, both the meningeal lymphatic drainage capability and MLV coverage were compromised in an Alzheimer’s disease (AD) mouse model, accompanied by cognitive impairment (Da Mesquita et al., 2018). Dysfunctions in both the glymphatic system and MLVs frequently co-occur in CNS diseases. Glymphatic system dysfunction often results in inadequate removal of metabolic waste from CSF through downstream MLVs, while disruption of MLVs hinders glymphatic clearance of ISF, leading to concurrent dysfunction between these systems (Da Mesquita et al., 2018; Li W. et al., 2022). For instance, traumatic brain injury (TBI) can impair AQP4 polarity and reduce glymphatic function by approximately 60% (Iliff et al., 2014). Concurrently, TBI can cause persistent MLV dysfunction for up to 1 month post-injury, indicating an interconnected pathology (Bolte et al., 2020). Besides, evidence for a simultaneous dysfunction of both glymphatic system and meningeal lymphatics in the context of chronic migraine conditions has been consolidated through non-invasive MRI imaging techniques (Wu et al., 2024). Analyses highlight an attenuated diffusion of water molecules surrounding the vasculature in individuals grappling with chronic migraines, in stark contrast to a control population manifesting healthy parameters. Investigations further reveal a decelerated fluid flow rate within the MLVs, culminating in a compromised effectiveness in CSF clearance, thereby convincingly illustrating a simultaneous dysfunction of both lymphatic systems (Table 1).

**TABLE 1:** Alterations in brain lymphatic system and manifestations in various CNS diseases.

Disease	Alterations in the brain lymphatic system	Blockage as an exacerbating factor	Associated pathological alterations and manifestations	Reference
PD	Reduced CSF influx and impaired glymphatic function	Yes	Accumulation of α-synuclein; Loss of dopaminergic neurons; Neuroinflammation; Motor deficits	Zou et al. (2019)
Idiopathic PD	Significant reduction in the flow of MLVs	yes	Motor and memory deficits; Accumulation of α-synuclein	Ding et al. (2021)
Traumatic brain injury	Severe impairment of meningeal lymphatic drainage	yes	Neuroinflammation; Cognitive deficits; Elevated ICP	Bolte et al. (2020)
AD	Decreased CSF influx and ISF efflux; Impaired MLV drainage	yes	Accumulation of Aβ; Cognitive deficits	Da Mesquita et al. (2018)
Chronic migraine	glymphatic and MLVs function impaired	N/A	Clinical disability and poor sleep quality	Wu et al. (2024)
Stroke	Meningeal lymphangiogenesis	yes	Larger infarct volume	Yanev et al. (2020)
Amyotrophic lateral sclerosis	Glymphatic system function impaired	N/A	Clinical disability and poor sleep quality	Liu et al. (2024)
Multiple sclerosis	Meningeal lymphangiogenesis near the cribriform plate	No	MLVs blockage reduces neuroinflammation	Hsu et al. (2019)

Abbreviations: PD, Parkinson’s disease; CSF, cerebrospinal fluid; MLVs, meningeal lymphatic vessels; ICP, intracranial pressure; AD, Alzheimer’s disease; ISF, interstitial fluid; Aβ, amyloid-β; N/A, not applicable.

Furthermore, in the presence of preexisting CNS diseases, impaired CNS lymphatic drainage often acts as an exacerbating factor. For instance, in an AD mouse model, inhibition of glymphatic system function via AQP4 knockout led to decelerated CSF inflow into the brain parenchyma, resulting in heightened accumulation of Amyloid-β (Aβ) and more pronounced cognitive deficits (Iliff et al., 2012). In a PD mouse model, dysfunction of glymphatic system drainage caused by ligation of the MLVs exacerbated α-syn accumulation, increased neuroinflammation, and worsened motor function impairment (Zou et al., 2019). In the context of stroke, mice with impaired MLVs often present with a larger infarct volume compared to wild-type mice (Yanev et al., 2020) (Table 1). When considering brain tumors, such as glioma and metastatic melanoma, obstruction of dorsal skull MLVs in mouse models diminishes the therapeutic effects of anti-Programmed Cell Death Protein 1/Cytotoxic T-Lymphocyte-Associated Protein 4 checkpoint combined therapy on these tumors (Hu et al., 2020). In an AD mouse model, ablation of MLVs intensifies AD pathology and cognitive performance decline by promoting Aβ accumulation, activating microglia proliferation, causing behavioral deficits and deterioration, while also reducing the effectiveness of anti-Aβ immunotherapy (Da Mesquita et al., 2018; Wang et al., 2019). Conversely, the latest research suggests that the blockade of MLVs function did not lead to an increase in the accumulation of pathological proteins in AD mouse models, nor did it exacerbate cognitive impairments (Antila et al., 2024). Such discrepancies may be attributed to variations in animal models, methodologies, and compensatory mechanisms of CSF dynamics. The complexity of CNS disease pathophysiology involves numerous factors and pathways, with various studies potentially revealing different aspects of this network. Therefore, further investigation of MLVs in the CNS diseases to clarify their role in disease pathology is needed.

The evidence presented indicates that dysfunction in the glymphatic system and meningeal lymphatic system is observed across a spectrum of neurological diseases, where compromised CNS lymphatic drainage frequently exacerbates disease severity, thus establishing a detrimental cycle. Therefore, the regulation of CNS lymphatic functions assumes paramount importance for the effective treatment of CNS diseases.

## 3 Glymphatic system-mediated therapeutic strategies for CNS disease

The glymphatic system, by means of its exchange between CSF and ISF, is integrally involved in the clearance of metabolic waste and the provision of nutrients within the brain (Figure 2). This crucial function is essential for maintaining cerebral health and preventing neurodegenerative diseases (Ding et al., 2023). Nonetheless, impairments in glymphatic functioning often coincide with various conditions, resulting in the obstruction of pathological macromolecules and hindered distribution of metabolic waste. As a result, intraneural accumulation occurs, subsequently contributing to the development of neurodegenerative diseases (Kress et al., 2014). Therefore, interventions targeting glymphatic system function through physiological factors, pharmacological modulation, and physical therapy hold substantial therapeutic potential.

### 3.1 Modulating physiological factors to intervene in the glymphatic system

#### 3.1.1 Sleep regulation

The role of sleep in eliminating metabolic waste from the brain is crucial. In fruit fly models of AD, sleep deprivation exacerbates the accumulation of Aβ, which shows an inverse correlation with the reported duration of nocturnal sleep (Shokri-Kojori et al., 2018). It has been demonstrated that during sleep, there is approximately a 60% increase in intercellular space, promoting enhanced convective flux of ISF within brain tissue and significantly improving CSF-ISF exchange (Xie et al., 2013). Furthermore, the glymphatic system in mice exhibits a pronounced diurnal rhythm where maintaining such rhythms relies on the polarization of AQP4 in the vascular endfeet of astrocytes as a fundamental factor (Hablitz et al., 2020). Moreover, during non-rapid eye movement sleep, the slow-wave neural activity can induce fluctuations in cerebral blood flow. These blood flow oscillations are coupled with the macroscopic oscillations of CSF, proving that neural activity can regulate the outflow of CSF (Fultz et al., 2019). This, in turn, indirectly affects the efficiency of the exchange between CSF and ISF within the glymphatic system, accelerating the clearance of metabolic waste. Thus, by modulating the sleep-wake cycle, it may be possible to harness the clearance functionality of the glymphatic system for improving neurodegenerative diseases.

#### 3.1.2 Alteration of body position

Different body positions during sleep also impact the function of the glymphatic system. Studies have discovered that the lateral position, or side-lying posture, is most effective in facilitating cerebral glymphatic transport and waste clearance, while the prone position demonstrates lower efficiency in waste removal (Lee et al., 2015). Furthermore, research has validated the influence of body position on glymphatic transport in humans. By utilizing upright MRI technology, images were acquired from 30 healthy volunteers in both standing and supine postures. It was observed that the CSF pulse volume during the cardiac cycle increased by approximately 57.6% in the supine position compared to standing (Muccio et al., 2021). This finding substantiates the notion that there is an enhanced CSF when one is a supine position, thus facilitating convection within the glymphatic system. This new perspective opens up an avenues for further exploration of the interplay between body position, brain-waste clearance, aging, and a series of neurodegenerative diseases. Additionally, it offers crucial insight for researchers: the influence of body position should not be overlooked when conducting imaging studies on glymphatic system functionality.

#### 3.1.3 Thermoregulation

Body temperature is an influential factor in the functioning of the glymphatic system and cannot be overlooked. Research indicates that early administration of hypothermic treatment can inhibit the velocity of CSF, thereby enhancing the immune response within the glymphatic system in a murine model of cerebral trauma (Li et al., 2024). This internal fluid process has been confirmed by high-temporal and spatial-resolution near-infrared two-region (NIR-II) imaging. Furthermore, hypothermic treatment upregulates the expression of genes associated with cerebral tissue repair (Li et al., 2024). Further investigation is required to determine whether this approach can improve pathological outcomes following cerebral injury (Ding et al., 2023).

#### 3.1.4 Physical exercise

Physical exercise plays a substantial role in promoting the clearance of metabolites through the glymphatic system, reducing neuroinflammation, and enhancing cognitive function. Studies have shown that voluntary exercise in aged mice improves spatial memory capabilities in water maze tests. This activity stimulates the expression of AQP4 protein and expedites solute macromolecule clearance within the glymphatic system, thereby reducing the deposition of Aβ (Maliszewska-Cyna et al., 2016; He et al., 2017). Furthermore, it has been further evidenced that exercise can mitigate the activation of microglial cells and astrocytes, highlighting its potential to alleviate neuroinflammation (He et al., 2017). Consequently, incorporating moderate exercise into treatment plans for patients with neurodegenerative diseases could serve as a strategic mechanism to mitigate pathological accumulation in AD while potentially fostering cognitive improvements.

Research has emphasized the role of cerebral artery pulse activity as a crucial driving force for the movement of CSF within and throughout the brain tissue. A reduction in arterial pulsation is associated with a slower pace of CSF-ISF exchange, thus slowing down the clearance of macromolecules, including Aβ and tau (Iliff et al., 2013). With aging, there is a decline in the efficiency of the glymphatic system, responsible for clearing large solute molecules, which is linked to reduced pulsation in cerebral artery walls (Iliff et al., 2012; Kress et al., 2014). Modulating these physiological factors that influence glymphatic system function may partly involve changes in cerebral artery pulsation penetrability. Furthermore, identifying novel physiological factors that regulate the penetrability of cerebral artery pulsations and promote CSF flow into brain tissue holds significant implications for clearing metabolic waste.

### 3.2 Pharmacological modulation of glymphatic system functionality

#### 3.2.1 Targeted pharmacological modulation

AQP4 is expressed on the foot processes of astrocytes, which form the outer walls of the perivascular space. This allows CSF to permeate the brain parenchyma, playing a crucial role in maintaining fluid balance within the brain’s barriers dynamically (Peng et al., 2023). Genetic deletion of AQP4 in murine models leads to a conspicuous reduction in CSF influx into the brain parenchyma (Mestre et al., 2018). In an AD mouse model characterized by tau pathology accumulation, both the CSF-ISF exchange of the glymphatic system and the polarization of AQP4 were impaired, with further exacerbation caused by TGN-02-induced pharmacological inhibition of AQP4. These findings support the notion that targeting AQP4 could be essential for eliminating waste from CNS diseases (Harrison et al., 2020). Therefore, rectifying alterations in AQP4 polarity through genetic or pharmacological means holds significant therapeutic potential for improving various CNS diseases.

Research has indicated that following an ischemic stroke, the decline in Dystrophin 71 (DP71) expression can lead to impaired AQP4 polarity, resulting in compromised function of the glymphatic system and hindering the clearance of vasogenic edema from ischemic sites (Yang et al., 2024). It has been discovered that a nucleic acid-based drug overexpressing DP71 can effectively improve brain edema by promoting co-localization and interaction between AQP4 and DP71, consequently restoring AQP4 polarity (Yang et al., 2024). Similarly, in a mouse model of PD, reduced CSF influx and efflux result in decreased efficiency of CSF-ISF exchange and compromised AQP4 polarity (Zou et al., 2019). Mechanistically, PD causes abnormal activation of Matrix Metalloproteinase-9 (MMP9), which triggers cleavage of β-dystroglycan, impacting AQP4 polarity and subsequently damaging the function of the glymphatic system (Fang et al., 2021; Si et al., 2024). Pharmacological inhibition of MMP9 using the inhibitor GM6001 not only restores the polarity of AQP4 and contacts between the basement membrane and astrocyte end-feet but also exhibits neuroprotective effects and promotes metabolic homeostasis in PD (Si et al., 2024). These findings suggest that inhibiting MMP-9 to restore AQP4 polarity could be a potent strategy for enhancing glymphatic system function in PD.

#### 3.2.2 Traditional pharmacological modulation

The modulation of glymphatic system function is acknowledged as a significant role played by traditional hormonal therapeutics and their antagonists (Yang et al., 2024). In the context of a TBI model, cerebral edema occurring alongside a norepinephrine storm presents challenges to glymphatic system functionality. The utilization of norepinephrine receptor antagonists (prazosin, propranolol, and atipamezole) has been observed to enhance CSF influx into brain tissue and effectively clear cellular debris therein. This mitigates tau accumulation, neuroinflammation, and alleviates cerebral edema (Hussain et al., 2023). Moreover, research has revealed that glucocorticoid dexamethasone (Sun et al., 2024) along with selective α2-adrenergic receptor agonist dexmedetomidine (Persson et al., 2022) fosters CSF influx into brain tissue and improves glymphatic system functionality. However, further investigation is required to determine the specific underlying mechanisms involved. It should be noted that invasive procedures, such as intracranial injections, can significantly disrupt glymphatic system function; thus they should be avoided when exploring its functionality (Mestre et al., 2018).

### 3.3 Physical therapy

Emerging studies suggest that gamma multisensory stimulation exerts regulatory effects on the glymphatic system and ameliorates the pathological manifestations of AD. Researchers have induced corresponding neural activities in certain brain areas through multisensory gamma stimulation, which enhances clearance functionalities within the glymphatic system, leading to a reduction in amyloid protein accumulation in AD mouse models (Murdock et al., 2024). This outcome is attributed to gamma stimulation, which promotes polarization of the water channel protein AQP4 in astroglial cells and expands MLVs. Moreover, it promotes glymphatic clearance by mediating pulsations in cerebral arteries through Vasointestinal Peptide (VIP) neuron-dependent activity (Nedergaard and Goldman, 2020). Repetitive transcranial magnetic stimulation (rTMS) has exhibited similar effects. Notably, early high-frequency rTMS treatment in AD mouse models effectively reduces long-term memory loss and mitigates AD-related pathological development, including Aβ deposition and glial cell activation, by enhancing the excretory efficiency of the glymphatic system (Lin et al., 2021). Consequently, gamma multisensory stimulation and rTMS, along with other physical therapies alike, represent promising therapeutic strategies for improving glymphatic system function, thereby ameliorating the prognosis of neurodegenerative diseases.

In summary, modulation of glymphatic system functionality holds significant value for the treatment of CNS diseases. The enhancement of this system’s capabilities through physiological regulation, pharmacological intervention, and physical therapy can facilitate more efficient clearance of detrimental substances within the brain parenchyma. This process may mitigate or prevent the onset and progression of neurodegenerative disorders.

## 4 Therapeutic strategies for modulating the meningeal lymphatic system to address CNS diseases

Abundant research indicates that dysfunction in the drainage of MLVs is observed in neurological disorders, and adverse MLV conditions may exacerbate a wide range of diseases, including neurodegenerative diseases such as AD and PD (Da Mesquita et al., 2018). Therefore, augmenting the functionality of MLVs is an important approach for delaying or preventing neurological diseases. Current methodologies encompass genetic techniques, pharmacological interventions, photobiomodulation therapy (PMT), and immunological therapies.

### 4.1 Targeted manipulation techniques for modulating MLVs

#### 4.1.1 VEGF-C

The potential role of VEGF-C in the enhancement of various neurological diseases has been extensively documented due to its reported ability to promote the development, formation, and maintenance of MLVs (Antila et al., 2017). Initially, direct administration of VEGF-C was employed for treating these conditions. In neurodegenerative diseases, impaired MLV function can hinder the exchange between CSF and ISF, leading to reduced clearance of pathological macromolecules and metabolic waste in the brain as well as cognitive impairment (Da Mesquita et al., 2018). Treatment with VEGF-C in elderly murine models has demonstrated an enhanced exclusionary effect on large molecules by MLVs within CSF, thereby strengthening the renewal and perfusion processes within intracranial CSF while subsequently improving learning and memory performance (Da Mesquita et al., 2018). In neurovascular disorders like stroke, studies have shown that the overexpression of VEGF-C enhances the formation of MLVs and meningeal lymphatic drainage, while reducing microglial activation and neuroinflammation. This mitigates motor function impairment, ameliorates the prognosis of ischemic stroke (Boisserand et al., 2024). Within the context of neuroinfectious diseases, researchers infected mouse models with Japanese encephalitis virus (JEV) and observed that post-CNS viral infection promotes neogenesis and expansion of MLVs but can also induce their dysfunction (Li X. et al., 2022). Photocoagulating MLVs or ligating draining lymph nodes in virus-infected mice exacerbates host’s neural tissue damage and increases mortality rate. However, gel-encapsulated VEGF-C recombinant peptide treatment appears to restore MLV function after application, reducing CNS damage caused by the virus, and enhancing survival rate (Li X. et al., 2022).

Additionally, indirect methods of increasing VEGF-C levels can be used for the treatment of brain diseases. Recent research has demonstrated that transplantation of cranial precursor cells in a murine model with premature cranial closure fosters the growth and development of MLVs, leading to the restoration of intracranial pressure (ICP), improved cerebral perfusion, and enhanced cognitive function. These beneficial effects are primarily attributed to the ability of cranial precursor cells to secrete VEGF-C (Ma et al., 2023). In conclusion, VEGF-C emerges as a potent therapeutic agent for safeguarding MLVs and holds promise for managing diverse neurological conditions. However, further investigations are warranted to ascertain their long-term therapeutic efficacy and overall safety.

#### 4.1.2 PIEZO1

The Piezo-Type Mechanosensitive Ion Channel Component 1 (PIEZO1) protein is an ion channel present on the cellular membrane, which transduces mechanical signals into electrochemical signals in response to mechanical stimuli, thereby facilitating intracellular signal transduction (Hill et al., 2022). It has been corroborated to be involved in endothelial cell genesis, proliferation, and repair processes (Zhang et al., 2022). In mouse models of premature cranial closure, increased ICP has been observed to affect CSF flow, consequently restricting the sprouting and expansion of MLVs (Aspelund and Alitalo, 2024). The employment of Yoda1 as a PIEZO1 agonist improves CSF flow dynamics, reduces ICP levels, promotes the growth and maintenance of MLVs, and enhances cerebral waste clearance as well as immune functionality (Matrongolo et al., 2023). The significant role of PIEZO1 has also been confirmed in mouse models of Down syndrome and hydrocephalus (Choi et al., 2024), where Yoda1 markedly alleviates symptoms related to excessive CSF accumulation and ventricular expansion. Therefore, considering its ability to regulate MLV growth and development effectively, the PIEZO1 agonist Yoda1 holds promising potential for future applications in modulating MLVs. However, further investigation is required to elucidate the specific mechanisms by which it regulates ICP levels and promotes MLV development while assessing its long-term therapeutic effects.

#### 4.1.3 DSCR1

The protein Down Syndrome Critical Region 1 (DSCR1) is characterized by its elevated expression in the cerebral tissues of individuals with Down syndrome on chromosome 21 (Baek et al., 2009). It is known to regulate angiogenesis and endothelial cell activation (Minami et al., 2009). Recent findings have also demonstrated its role as a regulatory factor in the development of MLVs (Choi et al., 2021). Investigations have revealed that overexpression of DSCR1 in AD mouse models promotes the development of dorsal MLVs, enhances the clearance of Aβ, and facilitates memory function. Mechanistically, DSCR1 exerts its effects through its downstream factor, Nuclear Factor of Activated T (NFAT) cells, which regulates the Wingless-Related Integration Site (Wnt) signaling pathway, thereby improving MLV function (Choi et al., 2021). Nevertheless, further exploration is required to elucidate the precise underlying mechanisms.

#### 4.1.4 CGRP

Calcitonin Gene-Related Peptide (CGRP), derived from unique RNA splicing forms of the calcitonin gene, plays a significant role in disease pathogenesis by inducing vascular dilation and regulating inflammatory responses (Russo and Hay, 2023). In the context of chronic migraine, CGRP signaling impacts disease severity through modulation of immunocyte interactions and CSF outflow in MLVs (Nelson-Maney et al., 2024). Researchers have identified Calcitonin-Receptor-Like Receptor (CALCRL) and Receptor Activity-Modifying Protein 1 (RAMP1) as specific receptors for CGRP, which mediate biological functionality through receptor-ligand-initiated signal transduction (Zhang et al., 2016; Bowen et al., 2023). Anti-CGRP therapy involving the knock-out of *Calcrl* and *Ramp1* genes has demonstrated efficacy in alleviating symptoms of chronic migraines by restoring CSF drainage function of MLVs (Nelson-Maney et al., 2024). However, further verification through clinical trials is needed to assess the effects of such anti-CGRP treatments on prolonged CSF outflow and related conditions.

#### 4.1.5 THBS1

Thrombospondin-1 (THBS1) is an extracellular matrix protein and a member of the thrombospondin family, playing crucial roles in angiogenesis regulation, cell-signal transduction facilitation, and immune regulation (Kale et al., 2021). In the context of SAH, evidence reveals that red blood cells (RBCs) drain from MLVs to cLNs. When MLVs are obstructed, they exhibit exacerbated neuroinflammation, subsequently worsening neural consciousness (Chen et al., 2020). Maintaining clear MLV drainage, thus, holds substantial significance for the prognosis of SAH. Researchers have employed single-cell RNA sequencing and spatial transcriptomics technology to identify a strong correlation between calgranulin (S100A6) and THBS1 with SAH prognosis. THBS1 binds with CD47, causing apoptosis in LECs through Signal Transducer and Activator of Transcription 3 (STAT3)/B-Cell Lymphoma 2 (Bcl-2) signaling pathway as a regulatory mechanism (Wang et al., 2023). Hence, disrupting THBS1 and CD47 complexes via genetic or pharmacological methods such as gene editing, RNA interference, protein degradation, or competitive drug inhibition presents a promising strategy to restore MLV functionality, leading to improved SAH prognosis.

#### 4.1.6 NET

Neutrophil extracellular traps (NETs) encompass web-shaped DNA structures released by activated neutrophils, which cannot be classified as intracellular proteins (Abaricia et al., 2021). Research has emphasized the significance of targeting NETs to regulate MLV functionality (Wang et al., 2021). NETs have been detected in brain tissue and cerebral microthrombi of patients with cerebrovascular diseases, showing an inverse correlation with patient prognosis (Kang et al., 2020; Denorme et al., 2022). In conditions of bacterial meningitis induced by pneumococcal infection, a high presence of NETs within brain tissue obstructs the exchange of CSF and ISF, as well as the inflow of CSF into cLNs. Treatment with DNase I to degrade NETs has shown improvements in the circulatory pathway between CSF-ISF-CSF-MLVs-dcLNs and amelioration of cerebral edema symptoms (Pavan et al., 2021). Complementary to this, another research team has identified similar complications related to NET-induced CSF circulation diseases in SAH and explored potential treatment options involving the administration of DNase I and anti-Ly6G antibodies. These treatments have led to enhanced CSF circulation, reduced formation of cerebral microthrombi, and favorable outcomes for mice prognosis (Hao et al., 2023). This team further investigated the mechanisms underlying the exacerbation of hydrocephalus following intraventricular hemorrhage caused by NETs dysfunctioning MLVs, impeding the flow of CSF and macromolecules into cLNs via MLVs. Mechanistically, the activation of lymphatic endothelial membrane protein Fractalkine Receptor (CX3CR1) by NETs triggers damage to lymphatic endothelia and the formation of thrombi within the MLVs (Zhang et al., 2024). However, further extensive exploration is warranted to elucidate the specific mechanisms underlying the interaction between NETs and CX3CR1. Specifically, researchers have found that genetically knocking out CX3CR1 and manipulating NET degradation with DNase I can effectively enhance lymphatic brain drainage and alleviate symptoms associated with hydrocephalus (Zhang et al., 2024). Therefore, targeting NETs through enzymatic degradation and genetic manipulation emerges as a promising therapeutic strategy for cerebral diseases.

### 4.2 Pharmacological regulation of MLVs

#### 4.2.1 Atorvastatin

Atorvastatin, a well-established lipid-lowering medication, has been demonstrated to foster the resorption of subdural hematoma while mitigating the inflammation induced by the hematoma (Li et al., 2014; Quan et al., 2019). Recent investigations have revealed that following subdural hemorrhage in rats, endothelial cells in MLVs exhibit injuries characterized by dilated luminal spaces and disrupted interconnections. The underlying mechanism involves induced dephosphorylation of Extracellular Signal-Regulated Kinases 1 and 2 (ERK1/2) in meningeal LECs. The efficacy of Atorvastatin in reversing dephosphorylation in LECs has been ascertained, ameliorating basal MLV drainage and facilitating the clearance of subdural hematomas (Yuan et al., 2024).

#### 4.2.2 Borneol

Borneol, a naturally occurring bicyclic monoterpene with high lipophilicity capable of crossing the blood-brain barrier (BBB), has recently been investigated (Li et al., 2012; Zhang et al., 2017). Upon oral administration of borneol microemulsion to mice, an increased clearance rate of Aβ from MLVs was observed, consequently facilitating the entry of large molecules into dcLNs and ameliorating the behavior and cognitive abilities in AD mice (Wu et al., 2023). Mechanistically, the application of borneol microemulsion was found to elevate the expression levels of Lymphatic Vessel Endothelial Hyaluronan Receptor 1 (LYVE-1), Forkhead Box C2 (FOXC2), and VEGF-C in the murine meninges, promoting the formation of MLVs while enhancing their diameter and permeability. Furthermore, borneol demonstrated its ability to decrease the levels of nitric oxide in the meninges, stimulating lymphatic vessel contraction and accelerating the clearance of Aβ (Wu et al., 2023). Hence, borneol emerges as a potential pharmacological agent capable of restoring MLV function and improving the prognosis for neurodegenerative diseases.

#### 4.2.3 Vitamin D

Vitamin D is recognized for its endothelial-protective properties (Schroder-Heurich et al., 2019). Liu et al. (2020) observed that subdural hematoma clearance involves drainage into dcLNs via MLVs. Subsequent to subdural hematoma formation, the development and functionality of MLVs are compromised, resulting in a reduced drainage rate and downregulation of lymphangiogenesis-associated molecular markers such as VEGF-C, LYVE1 and FOXC2. In a study by Chen et al. (2024), the administration of Vitamin D to mice with subdural hematomas significantly decreased hematoma volume while facilitating hematoma drainage to dcLNs via MLVs. After treatment with Vitamin D, an upregulation of PROX1, LYVE1, and other markers related to lymphatic vessel functionality was observed alongside a decrease in the expression of inflammatory factors Interleukin-6 (IL-6), IL-8, and Tumor Necrosis Factor α (TNF-α). Therefore, it can be concluded that Vitamin D promotes absorption of hematomas and exerts anti-inflammatory effects by preserving MLV integrity.

### 4.3 Photobiomodulation therapy

With the advancement of precision medicine, there is an increasing demand for non-invasive and non-traumatic treatment modalities (Salehpour et al., 2022). PMT, an emerging approach for neuroprotection, employs visible and near-infrared light to stimulate the biochemical activities of mitochondrial components, thereby modulating cellular processes (Wong-Riley et al., 2005). Consequently, researchers have utilized non-invasive near-infrared light treatments to mediate cerebral diseases by regulating the lymphatic system (Salehpour et al., 2022). For instance, by directing near-infrared irradiation upon MLVs based on their superficial distribution characteristics, investigators observed improved cognitive function and reduced Aβ deposition in AD mice. This was attributed mechanistically to PMT’s regulation of mitochondrial metabolism and cell conjunction in meningeal LECs (Zinchenko et al., 2019; Wang M. et al., 2024). Additionally, near-infrared light irradiation of MLVs at doses of 5 and 10 J/cm^2^ demonstrated increased permeability of the lymphatic endothelium, promoting relaxation of lymphatic vessels and enhancing clearance and drainage of macromolecules within the brain (Semyachkina-Glushkovskaya et al., 2020). Furthermore, accelerating the elimination of RBCs is of utmost importance in alleviating complications arising from intraventricular hemorrhage in preterm infants. Following low-level infrared light stimulation of MLVs, it was discovered that the removal RBCs within the ventricles was accelerated, leading to their drainage to dcLNs through MLVs. This process effectively reduces the toxic side effects associated with intraventricular RBC retention and enhances neural function recovery after hemorrhage (Li et al., 2023). While PMT targeting MLVs holds significant implications for the treatment of brain diseases, further exploration is warranted due to current limitations such as cranial scattering effects, laser wavelength optimization, irradiation duration adjustment, and potential side reactions.

### 4.4 Immunotherapy for brain diseases mediated by MLVs

In the exploration of immunotherapeutic strategies for brain diseases, a growing body of research has substantiated the integral role played by MLVs in this field (Schiller et al., 2021). Subsequent sections will elucidate the immunomodulatory functions of MLVs in various cerebral diseases and how these pathways can be targeted to develop innovative therapeutic strategies.

Initially focusing on MLV-mediated immunotherapy in cerebral neoplasms, accumulating evidence suggests that VEGF-C signaling plays a crucial role in enhancing immune surveillance of brain tumors (Song et al., 2020). By delivering VEGF-C mRNA through an adenovirus vector, researchers have successfully enhanced specific expression of VEGF-C within the brain, thereby eliciting adaptive immune responses in deep lymph nodes. This cascade facilitates the migration of CD8^+^ T cells towards the microenvironment of glioblastomas via MLVs, ultimately reshaping the immune microenvironment with the ultimate goal being tumor destruction (Song et al., 2020). Moreover, the efficacy of inhibiting glioblastoma growth can be significantly boosted by combining immune checkpoint inhibitors with VEGF-C mRNA treatment (Song et al., 2020), offering new strategic avenues for GBM therapy. Further investigations have indicated that the conjunction of VEGF-C mRNA therapy with radiotherapy - by expanding MLVs - promotes dendritic cell migration and activates CD8^+^ T cells, thereby amplifying radiotherapy-induced antitumor immune responses (Zhou et al., 2022). However, whether MLVs can enhance the release of tumor-specific antigens induced by radiotherapy necessitates additional scrutiny.

In contrast to malignancies, treatment strategies for autoimmune disorders need the prevention of immune cell activation and brain invasion (Hsu et al., 2019). Take experimental autoimmune encephalomyelitis (EAE), a mouse model of multiple sclerosis, for instance; disease pathogenesis is accompanied by the development of lymph vessels at the cribriform plate. These vessels facilitate the transportation of inflammatory signals from the CNS to cLNs, triggering adaptive immune responses (Hsu et al., 2019). By utilizing MAZ51, a VEGFR3 tyrosine kinase inhibitor that hinders VEGFR3-dependent lymphangiogenesis and eliminates MLVs, researchers have observed a reduction in the severity of EAE and suppression of inflammatory reactions by brain-reactive T cells (Hsu et al., 2019; Li G. et al., 2022). Nevertheless, the long-term implications of MLV blockade on overall health continue to prompt further investigations. In addition, researchers have initiated a clinical trial (NCT05414487) utilizing Ofatumumab as an immunomodulatory drug for treating multiple sclerosis patients to explore its impact on MLVs and dynamic changes in immune cells. Consequently, scientists have dedicated significant efforts towards elucidating strategies for autoimmune disease treatment involving MLVs, yet additional research is still required.

In neurodegenerative diseases, such as AD, the immunological role mediated by MLVs remains crucial. Elderly mice often demonstrate functional impairment of MLVs, leading to the accumulation of misfolded proteins and metabolic waste within the brain parenchyma. This accumulation triggers neurodegenerative diseases (Da Mesquita et al., 2018). Rectifying this age-related dysfunction in older mice represents a promising therapeutic target for managing neurodegenerative diseases. Through single-cell RNA sequencing, researchers have identified prominent alterations in meningeal immunity among elderly mice. These alterations are characterized by a notable increase in Interferon γ (IFN-γ) expression primarily attributed to activated CD4^+^ T cells and CD8^+^ cytotoxic T cells, which ultimately affects CSF drainage through MLVs (Rustenhoven et al., 2023). Therefore, it has been demonstrated that the drainage function of MLVs can be significantly improved by peripherally administering anti-IFNγ neutralizing antibodies, verifying the reversibility of age-related MLV impairment (Rustenhoven et al., 2023).

In conclusion, MLVs are observed to play a multifaceted and intricate role in the therapeutic management of cerebral diseases. By fostering an in-depth comprehension of the functionality and regulatory mechanisms of MLVs under varying disease states, there lies potential for developing more precise and efficacious strategies to improve the quality of life for individuals diagnosed with brain diseases. Notably, VEGF-C has been recognized for its significant potential clinical translation among these therapeutic approaches. Copious research reports (Salvador et al., 2024), suggest that VEGF-C, as a well-established lymphangiogenic factor, stimulates growth and development of the lymphatic system and promotes MLVs’ drainage, consequently improving CNS disease pathology and clinical manifestations. However, transitioning these findings to clinical practice may likely necessitate additional scrutiny to ascertain the optimal therapeutic strategies, dosage, and safety.

## 5 Therapeutic drug delivery mediated by the CNS lymphatic system

### 5.1 Drug delivery mediated by MLVs

The existence of the BBB serves as a significant hindrance to the treatment of CNS diseases (Terstappen et al., 2021). MLVs, serving as conduits connecting the peripheral lymphatic system with the CNS (Aspelund et al., 2015), offer a potential pathway for delivering drugs to the intracranial space. Drug delivery through this route may hold the prospect of precision therapy for CNS diseases. In the treatment of glioblastoma, the injection of Poly(lactic-co-glycolic acid) (PLGA) nanoparticles loaded with Indocyanine Green (ICG) near the cLNs, it was found that the nanoparticles entered the MLVs, which then resulted in a 44-fold higher uptake of the nanoparticle drugs in the brain than conventional intravenous injection. The excitation of ICG by near-infrared light for photodynamic treatment of tumors demonstrated that the residual nanoparticles significantly inhibited glioblastoma growth (Zhao et al., 2020). Additionally, immune cell membranes have also been utilized to encapsulate drugs for delivery via MLVs to treat CNS diseases. For instance, curcumin encapsulated within natural killer cell membranes has been utilized in PD, and it was discovered that the encapsulated drug colocalized with MLVs marked by Lyve-1, with a marked enrichment observed within the brain (Liu et al., 2023). Additionally, research has demonstrated that Fibroblast Growth Factor-21 (FGF21), a neuroprotective factor (Chen et al., 2019), when encapsulated in BV2 cell membranes, similarly exhibits the ability to traverse the MLVs and target areas affected by AD. This approach has been shown to reduce the accumulation of tau protein and ameliorate neuroinflammation (Wang H. C. et al., 2024). Therefore, drug delivery mediated by the MLVs offers a novel approach for the treatment of CNS diseases; however, its feasibility and the underlying mechanisms necessitate further experimental validation.

### 5.2 Drug delivery mediated by the glymphatic system

In addition to transporting drugs from peripheral to intracranial locations, the conveyance of drugs from the CSF to the deep brain parenchyma also has significant implications. Studies have shown that systemic injections of hypertonic saline can enhance the binding of anti-Aβ antibodies to Aβ in AD mouse models, owing to a reduction in brain tissue volume caused by elevated plasma osmotic pressure. Interestingly, this effect enlarges the perivascular space and enhances glymphatic system function while leaving brain arterial volume unaffected (Plog et al., 2018). Similar effects of hypertonic saline have been substantiated in spinal drug treatments as well (Blomqvist et al., 2022). Furthermore, researchers have initiated investigations aiming to utilize the glymphatic system for directing nanoparticles from CSF into deep brain parenchymal regions. It has been established that following systemic injection of hypertonic saline and intrathecal administration of radioactively labeled gold nanoparticles, both single-photon emission computed tomography and MRI detected extensive distribution of nanoparticles within deep brain tissue regions, with rapid renal metabolic clearance occurring within 24 h (Lilius et al., 2023). Leveraging the perivascular space in the glymphatic system as a pathway for delivering immunopharmacological agents or nanodrugs into deep brain parenchymal regions presents a promising approach. These insights lay a solid foundation for new strategies involving intrathecal drug delivery assisted by the glymphatic system.

## 6 Discussion

This review proffers an overview of the therapeutic potential and challenges associated with applying the CNS lymphatic system in CNS diseases. Initially, a historical perspective on the discovery of the CNS lymphatic system is revisited, along with an examination of the anatomical features of the glymphatic system and MLVs, highlighting their crucial roles in waste clearance and immunological regulation. Dysfunctions within these systems are intrinsically linked to an array of CNS disorders, including AD and PD, among others. Subsequently, strategies aimed at modulating brain diseases are explored by enhancing the functionality of the CNS lymphatic system through adjustments in physiological factors, pharmaceutical interventions, and physical therapy methods. Lastly, the unique utility of the CNS lymphatic system in drug delivery provides novel strategies for circumventing BBB. Nevertheless, further research in this field is still in its early stages, necessitating additional experimental and clinical studies to confirm the clinical applicability of these findings.

The CNS lymphatic system holds significant therapeutic potential for intervening in CNS diseases. Despite extensive research efforts dedicated to investigating its physiological function, pathological mechanisms, and strategies for disease treatment, certain limitations persist that demand solutions. In terms of anatomical structure, it remains unclear whether MLVs exist in the pia mater and if their structure can be revealed using current electron microscopy technology. From a mechanistic standpoint, further investigation is required to elucidate the precise functional mechanisms of the CNS lymphatic system in CSF circulation, metabolic waste clearance, and immune regulation though non-invasive high-resolution imaging techniques. Addressing these inquiries would establish a theoretical foundation for implementing treatment strategies. Moreover, identifying novel therapeutic targets within the CNS lymphatic system is of significant importance for the development of new treatment approaches. Examination of applications suggests that further development of novel therapeutic strategies for CNS diseases, such as integrating nanotechnology and chemical synthesis technology in MLV-mediated nano-drug delivery, is warranted. Whereas, it is important to note the potential variability in therapeutic efficacy due to inter-individual differences in the CNS lymphatic system. Urgent questions remain regarding the differential role of the CNS lymphatic system under various disease conditions and how to personalize treatment plans according to individual variances.

Apart from mechanistic aspects of the CNS lymphatic system that beckon further exploration, there are inherent challenges within its clinical applications that must be overcome. Primarily, interventions in the CNS lymphatic system require rigorous examination through increased pre-clinical and clinical research to thoroughly evaluate the long-term safety and efficacy of these treatments. Furthermore, identifying optimal dosage for these therapies may present significant challenges, given that medication doses derived from animal models do not directly apply to humans. Patients may exhibit varied responses to treatments, differentiated drug absorption, and varied metabolic rates, necessitating the deployment of stringent, multi-centered, large-scale clinical trials to evaluate the medicinal dosage against corresponding efficacy. Notably, these therapeutic modalities frequently entail drug delivery challenges, given the unique characteristics of CNS disease sites, including the impediments posed by the BBB and the blood-cerebrospinal fluid barrier. The methodologies employed in animal models, such as craniotomy for the administration of therapeutics, are often not feasible for translation to human applications. Consequently, there is an imperative need to develop drug delivery systems that are tailored for human use, particularly for the delivery of labile genetic and nucleic acid therapeutic agents, which are susceptible to degradation. Research at present largely remains confined to the animal level, evidencing a glaring dearth of direct human study, notably in the relation between meningeal lymphatic function and human cognitive function.

In conclusion, the study of the CNS lymphatic system has not only augmented our comprehension of CNS diseases but also engendered potential for innovative treatment strategies. Future research agendas necessitate overcoming existing limitations, delving deeper into the roles and mechanisms of the CNS lymphatic system, as well as developing therapeutic strategies for CNS diseases. It is highly anticipated that continuous research and innovation will lead to more efficacious treatment modalities emerging for patients burdened with CNS afflictions.
